# CRISPR-aided genome engineering for secondary metabolite biosynthesis in *Streptomyces*

**DOI:** 10.1093/jimb/kuae009

**Published:** 2024-03-04

**Authors:** Yongjae Lee, Soonkyu Hwang, Woori Kim, Ji Hun Kim, Bernhard O Palsson, Byung-Kwan Cho

**Affiliations:** Department of Biological Sciences, Korea Advanced Institute of Science and Technology, Daejeon 34141, Republic of Korea; Department of Biological Sciences, Korea Advanced Institute of Science and Technology, Daejeon 34141, Republic of Korea; Department of Biological Sciences, Korea Advanced Institute of Science and Technology, Daejeon 34141, Republic of Korea; Department of Biological Sciences, Korea Advanced Institute of Science and Technology, Daejeon 34141, Republic of Korea; Department of Bioengineering, University of California San Diego, La Jolla, CA 92093, USA; Department of Pediatrics, University of California San Diego, La Jolla, CA 92093, USA; Novo Nordisk Foundation Center for Biosustainability, Technical University of Denmark, Lyngby 2800, Denmark; Department of Biological Sciences, Korea Advanced Institute of Science and Technology, Daejeon 34141, Republic of Korea; KAIST Institute for the BioCentury, Korea Advanced Institute of Science and Technology, Daejeon 34141, Republic of Korea; Graduate school of Engineering Biology, Korea Advanced Institute of Science and Technology, Daejeon 34141, Republic of Korea

**Keywords:** *Streptomyces*, CRISPR/Cas, biosynthetic gene cluster

## Abstract

The demand for discovering novel microbial secondary metabolites is growing to address the limitations in bioactivities such as antibacterial, antifungal, anticancer, anthelmintic, and immunosuppressive functions. Among microbes, the genus *Streptomyces* holds particular significance for secondary metabolite discovery. Each *Streptomyces* species typically encodes approximately 30 secondary metabolite biosynthetic gene clusters (smBGCs) within its genome, which are mostly uncharacterized in terms of their products and bioactivities. The development of next-generation sequencing has enabled the identification of a large number of potent smBGCs for novel secondary metabolites that are imbalanced in number compared with discovered secondary metabolites. The clustered regularly interspaced short palindromic repeat (CRISPR)/CRISPR-associated (Cas) system has revolutionized the translation of enormous genomic potential into the discovery of secondary metabolites as the most efficient genetic engineering tool for *Streptomyces*. In this review, the current status of CRISPR/Cas applications in *Streptomyces* is summarized, with particular focus on the identification of secondary metabolite biosynthesis gene clusters and their potential applications.

This review summarizes the broad range of CRISPR/Cas applications in *Streptomyces* for natural product discovery and production.

**One-Sentence Summary:**

This review summarizes the broad range of CRISPR/Cas applications in *Streptomyces* for natural product discovery and production.

## Graphical Abstract

**Graphical Abstract:**
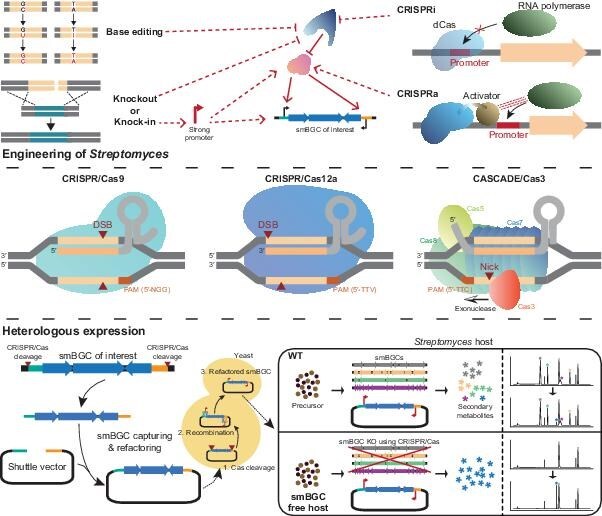

## Introduction

Most of the currently used antibiotics were discovered during the “golden era of antibiotic discovery” spanning from the 1940s and 1960s (Lewis, 2013). Although novel types of antibiotics have rarely been discovered since that period, the demand for novel antibiotics has increased to address the escalating threat posed by multi-drug-resistant pathogens (Boucher et al., 2009). The genus *Streptomyces*, consisting of a large number of species, holds significance as a major antibiotic producer. Approximately two-thirds of the clinically used antibiotics have been discovered in this genus. In addition, their secondary metabolites often possess additional beneficial activities related to medical, agricultural, and chemical applications, such as antitumorals and anthelmintics (Arcamone et al., 1969; Burg et al., 1979).

As an abundant reservoir of secondary metabolites, a single *Streptomyces* species is expected to encode approximately 30 secondary metabolite biosynthetic gene clusters (smBGCs) in its genome. However, the characterization of the products synthesized from many of the smBGCs has been limited (Lee et al., 2020a). Considering the abundance of species within the *Streptomyces* genus and the ease of isolating novel species, the discovery of at least tens of thousands of novel smBGCs is anticipated, providing a potential breakthrough in meeting the growing demand for novel antibiotics that are effective against multidrug-resistant pathogens (Khattab et al., 2016; Lee et al., 2020b). Although genome mining of streptomycetes reveals the existence of numerous unexplored bioactive compounds in the genomes, the actual discovery of novel secondary metabolites is far behind their potential, as their secondary metabolism undergoes complex regulation, and smBGCs often remain silent under laboratory culture conditions (Craney et al., 2013; Lee et al., 2020c). Therefore, there is an urgent need to activate silent smBGCs to fully harness the genomic potential of *Streptomyces*.

Multiple approaches have been used to activate silent smBGCs in *Streptomyces*. One such approach involves indirectly activating smBGCs by mimicking the environmental signals required for activation. For example, the one strain–many compound approach diversifies cultivation parameters, including media composition, aeration, temperature, and enzyme inhibitors, for a single strain to elicit secondary metabolism (Bode et al., 2002). Cocultivation with other organisms can also activate secondary metabolism (Marmann et al., 2014). However, these indirect approaches may result in the repetitive activation of the same smBGC in each trial. In addition, further efforts are required to identify the smBGCs encoded in the genome responsible for the biosynthesis of newly produced secondary metabolites.

Targeted activation of uncharacterized smBGCs is the preferred approach for the discovery of novel secondary metabolites, which involves engineering cluster-situated regulators, refactoring the target smBGC, and/or heterologous expression of the target smBGC (Baral et al., 2018). Efficient genetic manipulation tools are required for targeted smBGC activation. Fortunately, efficient clustered regularly interspaced short palindromic repeat (CRISPR)/CRISPR-associated (Cas)-mediated genome editing tools are available for *Streptomyces*, which have promoted the discovery of secondary metabolites (Tong et al., 2020). In this review, the current applications of the CRISPR/Cas system in *Streptomyces* are briefly described and discuss the potential of the CRISPR/Cas system to boost natural product discovery.

## CRISPR/Cas Systems Established in *Streptomyces*

### CRISPR/Cas System for *Streptomyces* Genome Engineering

**Fig. 1.**
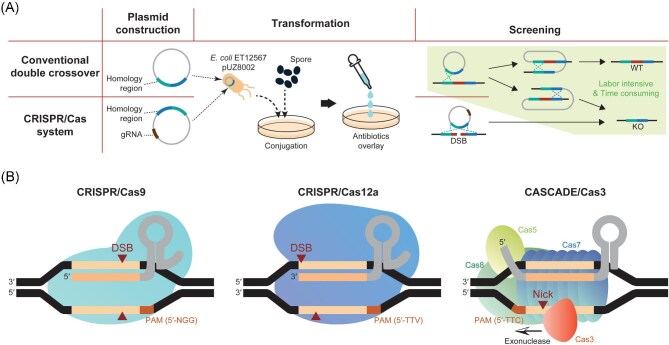
Genome engineering of *Streptomyces* using CRISPR/Cas system. (A) Comparison of gene deletion processes between CRISPR/Cas-mediated engineering and conventional double crossover-mediated engineering. (B) Types of CRISPR/Cas system exploited in *Streptomyces*.

The CRISPR/Cas system usually consists of two components: a Cas protein with endonuclease activity and a guide RNA containing a spacer sequence that directs the endonuclease to its complementary protospacer sequence; a scaffold facilitates this interaction (Jinek et al., 2012). The Cas endonuclease induces double-stranded DNA breaks (DSB) through two nuclease domains, HNH (complementary strand) and RuvC-like (non-complementary strand), only when the correct protospacer adjacent motif (PAM) is present, which precisely targets the CRISPR/Cas complex to the protospacer. Genome engineering using CRISPR/Cas involves the repair of DSB via either non-homologous end-joining (NHEJ) or homology-directed repair (HDR). During DNA repair via the NHEJ pathway, small insertions or deletions occur randomly at the DNA cleavage site (Doudna & Charpentier, 2014). In contrast, precise genome editing is enabled through DNA repair via the HDR pathway, which requires the donor DNA to be recombined into the cleavage site. Genes involved in the bacterial NHEJ pathway are absent in most *Streptomyces* species, and thus, genome editing mediated by the HDR pathway is preferred for CRISPR/Cas-based genetic engineering (Tong et al., 2015). Conventionally, achieving precise and scarless genome editing in *Streptomyces* was mostly dependent on double-crossover events, which required time-consuming and labor-intensive screening steps (Kieser et al., 2000). Given the lethal DSB induced by the CRISPR/Cas system in bacteria unless repaired, the surviving cells after transformation of the CRISPR/Cas plasmid are more likely to be successfully engineered. Consequently, the time and labor required for the screening steps are dramatically reduced (Fig. 1A) (Cobb et al., 2015; Tong et al., 2015).

The first CRISPR/Cas system applied to *Streptomyces* was based on the class 2–type II CRISPR system, which uses Cas9 from *Streptococcus pyogenes* as a nuclease (Fig. 1B) (Cobb et al., 2015). Cas9 requires 5′-NGG-3′ as the PAM, which is frequently found in the GC-rich *Streptomyces* genomes. Using the CRISPR/Cas9 system, genomic deletions of up to 31.4 kbp have been performed in three *Streptomyces* species: *S. lividans, S. viridochromogenes*, and *S. albus* (Cobb et al., 2015). Around the same period, additional CRISPR/Cas9 systems were explored, leading to additional applications and advancements (Huang et al., 2015; Tong et al., 2015; Zeng et al., 2015). To enhance genome editing efficiency via the NHEJ pathway, DNA ligase D, a core component of the NHEJ pathway, was co-expressed with the CRISPR/Cas system (Tong et al., 2015). Although the mutations induced by NHEJ-mediated DNA repair are random, the enhanced editing efficiency can facilitate rapid gene disruption as there is no requirement for donor DNAs for homologous recombination. In addition, although most CRISPR/Cas9 systems rely on a temperature-sensitive origin to cure the plasmid, an alternative counterselection method for screening plasmid-cured strains has been described (Zeng et al., 2015). A mutant cytosine deaminase, which converts 5-fluorocytosine into the highly toxic 5-fluorouracil, was integrated into the CRISPR/Cas9 plasmid, and treatment with 5-fluorocytosine effectively eliminates the strains retaining the plasmid (Zeng et al., 2015).

The CRISPR/Cas system for *Streptomyces* is gradually evolving by expanding its applicable hosts, including *S. rimosus, S. ambofaciens*, and *S. roseosporus*, and exploiting different types of CRISPR/Cas systems (Jia et al., 2017; Li et al., 2018; Najah et al., 2019; Yeo et al., 2019; Jiang et al., 2021). Especially, the class 2–type V CRISPR/Cas system, exploiting Cas12a from *Francisella novicida*, was applied to *Streptomyces*, expanding the number of editable DNA sequences with 5′-TTV-3′ (V = A, G, or C) PAM (Fig. 1B) (Li et al., 2018). Although the 5′-NGG-3′ PAM of Cas9 may provide a wide range of potential editing targets, the AT-rich PAM of Cas12a provides advantages for target specificity in the GC-rich genome of *Streptomyces*. In addition, Cas12a is more suited for multiplexing compared to Cas9, because it enables the maturation of CRISPR RNA (crRNA) (Zetsche et al., 2017).

In addition to expanding the PAM sequence, the establishment of different types of CRISPR/Cas systems for engineering *Streptomyces* is important because the exploration of secondary metabolites spans the entire genus, and the applicability of a specific type of CRISPR system may differ from species to species (Li et al., 2018; Yeo et al., 2019). Both CRISPR/Cas9 and CRISPR/Cas12a are convenient to use because only a single Cas protein is required to induce DSB in the genome (Pickar-Oliver & Gersbach, 2019). However, the introduced foreign CRISPR/Cas system may crosstalk with the native CRISPR/Cas system encoded within the *Streptomyces* genome, resulting in reduced engineering efficiency and severe defects in the host (Qiu et al., 2016). In particular, the class 1 type I CRISPR/Cas system, which requires a Cas3 nuclease and a ribonucleoprotein complex comprising Cas5, Cas7, Cas8 proteins, and crRNA, is the most widespread CRISPR/Cas system in *Streptomyces*. Thus, class 1 type I CRISPR/Cas could be advantageous for a broad range of *Streptomyces* species (Fig. 1B) (Whitford et al., 2023a).

### Utilization of Mutated Cas Proteins for *Streptomyces* Genome Engineering

Although the CRISPR/Cas system is efficient (Fig. 1A), there are some shortcomings associated with its application in the genome engineering of *Streptomyces*. In addition to inherent cytotoxicity, genome engineering using the CRISPR/Cas system is accompanied by DSB, which may induce severe instability in the linear genome of *Streptomyces* (Hoff et al., 2018). To reduce the lethality occurring from DSB, Cas9 variant nicking DNA (nCas), rather than cleaving double-stranded DNA, can be used to edit the genome (Ma et al., 2023). Genetic engineering using nCas variants is particularly effective for strains with low transformation efficiency. For example, the exploitation of nCas9 resulted in the recovery of hundreds more colonies from the transformation of an industrial *Streptomyces* strain, compared to the below 10 colonies when conventional Cas9 system was applied (Ma et al., 2023).

**Fig. 2.**
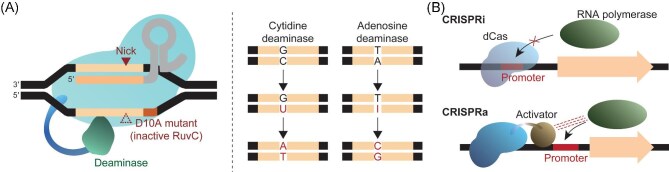
Utilization of mutated Cas proteins for genetic engineering of *Streptomyces*. (A) CRISPR base editors exploiting nCas9 and deaminase. (B) Transcriptional repression and activation exploiting dCas9.

An additional drawback of the CRISPR/Cas system may not be suitable for inducing point mutations as the guide RNA can still bind to the edited genome with a single mismatch (Zheng et al., 2017). To overcome this potential limitation, cytidine or adenosine deaminase is fused with nCas9 to form the base editor with high specificity for the target sequence driven by the CRISPR/Cas system (Fig. 2A) (Tong et al., 2019). Deaminated cytidine or adenine are eventually substituted with thymidine or guanosine, respectively, through a nucleotide excision repair process. Base editors can disrupt a start codon or introduce a stop codon to effectively generate a knockout mutant (Tong et al., 2019; Wang et al., 2020a). For example, a point mutation generated by a base editor facilitates the understanding of translational regulation in *Streptomyces*, such as the important regulatory role of the rare leucine codon UUA, decoded by a specific tRNA, *bldA*, in the development and secondary metabolism (Chater & Chandra, 2008).

The RNA-guided DNA binding ability of the CRISPR/Cas system has a broad range of applications. In addition to editing the genome sequence, the CRISPR/Cas system can be used to modulate gene expression via CRISPR interference (CRISPRi) and activation (CRISPRa) in *Streptomyces* (Fig. 2B) (Tong et al., 2015; Li et al., 2018; Ameruoso et al., 2022). Nuclease-dead Cas (dCas) variants can prevent the binding of transcriptional machinery to DNA or serve as roadblocks for transcribing RNA polymerase (Larson et al., 2013). In addition, dCas can be fused with a transcriptional activator such as the N-terminal domain of the RNA polymerase α subunit, to promote transcription in the vicinity of the guide RNA targeted region (Maeder et al., 2013; Ameruoso et al., 2022).

## CRISPR/Cas Applications for Natural Product Research in *Streptomyces*

### Characterization of Smbgcs Using the CRISPR/Cas System

Owing to their unparalleled convenience, CRISPR/Cas-based engineering tools are rapidly replacing conventional tools, finding broad applications in diverse aspects of natural product research, from the characterization of smBGCs to the enhancement of secondary metabolite production. For example, formicamycins, which show antimicrobial activity against methicillin-resistant *Staphylococcus aureus* and vancomycin-resistant enterococci, have been discovered in the *Streptomyces* isolate *S. formicae* KY5 (Qin et al., 2017). To identify the responsible smBGC, the entire region of the type 2 polyketide synthase (PKS) BGC was deleted using CRISPR/Cas9, resulting in the inactivation of formicamycin production. This strategy has been applied to identify smBGCs for multiple secondary metabolites, including sceliphrolactam, valinomycin, and griseusin, by deleting a synthesis gene in the smBGC or introducing a stop codon in the synthesis gene using the CRISPR/Cas system or base editor, respectively (Low et al., 2018; Jeon et al., 2019; Beck et al., 2021). Furthermore, the ease of smBGC disruption enables functional screening of smBGCs. In *S. globisporus* SP6C4, the biosynthetic genes of 15 smBGCs were deleted using the CRISPR/Cas system, and the smBGC-disrupted strains were tested for antifungal and antibacterial activities, revealing three bioactive lantipeptide- or lassopeptide-type secondary metabolites (Kim & Kwak, 2021).

The CRISPR/Cas system enabled not only the identification of the smBGC responsible for secondary metabolite production but has also played a key role in the elucidation of their biosynthetic pathway. For example, three genes in the anthelvencin BGC were deleted using the CRISPR/Cas9 system, and their role in anthelvencin synthesis was confirmed (Lee et al., 2021). This approach has also been applied to other secondary metabolites, including neoantimycin and hygromycin B, to characterize the functions of genes during secondary metabolite biosynthesis (Skyrud et al., 2018; Li et al., 2020a). In addition to the genes directly involved in secondary metabolite synthesis, genes affecting secondary metabolism outside smBGCs have also been characterized using the CRISPR/Cas system (He et al., 2016; Li et al., 2020b; Lee et al., 2020d; Hou et al., 2021). For example, the redox-sensitive regulator Rex was deleted in *S. lincolnensis*, and the deletion mutant produced a lower amount of lincomycin, suggesting that Rex is involved in the regulation of lincomycin biosynthesis (Hou et al., 2021). Similarly, the CRISPR/Cas system is innovative for characterizing smBGCs because of its high precision and speed, and is likely to accelerate the discovery of novel secondary metabolites using base editors.

### Utilization of the CRISPR/Cas System for Secondary Metabolite Production

The ultimate goal of engineering *Streptomyces* is the production of large amounts of valuable natural products. Secondary metabolite production can be enhanced by controlling the expression of biosynthetic genes or modulating regulatory genes related to the biosynthetic pathway of the target molecule (Wang et al., 2020b; Li et al., 2022). To elevate the expression of the target smBGC, various strategies can be applied, including deletion of negative regulators, overexpression of positive regulators, refactoring of the promoters of biosynthetic genes, and multiplying the copy number of the smBGC (Table 1).

**Table 1.** *In vivo* Applications of CRISPR/Cas System for Enhancement of Secondary Metabolite Production

`Strategy	Cas protein	Engineered species	Target secondary metabolite	Engineering	Year	Reference
Knock-in	Cas9	*S. pristinaespiralis*	Pristinamycin II	Knock-in of attB integration sites for smBGC copy number increasement	2017	(Li et al., 2017)
	Cas9	*S. coelicolor*	Chloramphenicol			
	Cas9	*S. coelicolor*	YM-216 391			
	Cas9	*S. coelicolor*	Undecylprodigiosin		2017	(Liu et al., 2017)
	Cas9	*S. albidoflavus*	Eriodictyol	Knock-in of genes for precursor supply	2023	(Magadán-Corpas et al., 2023)
	Cas9	*S. roseosporus*	Unknown type I PKS	Promoter knock-in of biosynthetic genes or a positive regulator	2017	(Zhang et al., 2017)
	Cas9	*S. albus*	Indigoidine			
	Cas9	*S. coelicolor*	Actinorhodin			
	Cas9	*S. coelicolor*	Undecylprodigiosin			
	Cas9	*S. roseosporus*	Polycyclic tetramate macrolactam			
	Cas9	*S. roseosporus*	FR-900 098			
	Cas9	*S. venezuelae*	Unknown type III PKS	Promoter knock-in of biosynthetic genes		
	Cas9	*S. viridochromogenes*	Unknown type II PKS			
	Cas9	*S. roseosporus*	Auroramycin		2018	(Lim et al., 2018)
	Cas9	*Streptomyces* sp. AL2110	Deacyl-antimycin		2019	(Mo et al., 2019)
	Cas9	*Streptomyces* sp. A793	Armeniaspirols		2023	(Heng et al., 2023)
	Cas12a	*S. roseosporus*	Daptomycin		2020	(Zhang et al., 2020)
Deletion	Cas9	*S. fradiae*	Neomycin	Deletion of a repressor	2019	(Zheng et al., 2019)
	Cas9	*S. lincolnensis*	Lincomycin		2023	(Wang et al., 2023b)
	Cas9	*S. roseosporus*	Daptomycin	Deletion of genes of competitive pathway	2022	(Ji et al., 2022)
	Cas9	*S. albidoflavus*	Eriodictyol		2023	(Magadán-Corpas et al., 2023)
	Cas9	*Streptomyces* sp. A793	Armeniaspirols		2023	(Heng et al., 2023)
	Cas9	*S. pristinaespiralis*	Pristinamycin I	Deletion of genes of competitive pathway and a repressor	2017	(Meng et al., 2017)
CRISPRi	dCas9	*S. venezuelae*	Pikromycin	Repression of a gene negatively affecting secondary metabolite production	2022	(Cho et al., 2022)
	dCas9	*S. rapamycinicus*	Rapamycin	Repression of genes of competitive pathway	2020	(Tian et al., 2020)
	dCas9	*S. venezuelae*	Jadomycin B	Repression of negative regulator	2022	(Ameruoso et al., 2022)
CRISPRa	dCas9-αNTD	*S. venezuelae*	Jadomycin B	Activation of biosynthetic genes		

In smBGCs, cluster-situated regulators often positively or negatively regulate the transcription of biosynthetic genes, which are attractive engineering targets. For example, *neoI*, a negative regulator of neomycin biosynthesis, was deleted to enhance neomycin production (Zheng et al., 2019). Furthermore, CRISPRi can be used to repress inhibitors and eventually activate the transcription of biosynthetic genes (Ameruoso et al., 2022). The expression of *jadR2*, a repressor of the positive regulator of jadomycin biosynthesis, was reduced by CRISPRi, resulting in the induction of jadomycin production in *S. venezuelae*. In the case of positive regulators, the upstream region of *aurR1*, a positive regulator of auroramycin BGC, was engineered by employing the constitutive kasO* promoter to activate transcription of biosynthetic genes in *S. roseosporus* (Lim et al., 2018). Modulation of regulatory genes represents an efficient strategy to transcriptionally activate smBGCs, as engineering only one target is required; however, secondary metabolism is subject to complex regulation, and multiple regulators may regulate smBGCs, limiting the effectiveness of the engineered regulator (Liu et al., 2019a). Engineering the promoters of secondary metabolite biosynthetic genes enables not only transcriptional activation but also the construction of an orthogonal regulatory network for the targeted smBGC. The promoter replacement of biosynthesis genes using the CRISPR/Cas system has been used to enhance secondary metabolite production or activate transcriptionally silent smBGCs in multiple *Streptomyces* species (Zhang et al., 2017; Mo et al., 2019).

Increasing the copy number of smBGC can significantly enhance secondary metabolite production. To this end, phage site-specific recombination systems are commonly utilized rather than directly inserting the entire smBGC using the CRISPR/Cas system (Li et al., 2017). The *attB* integration sites are first inserted into the genome of *Streptomyces* using the CRISPR/Cas system, and the plasmid bearing the target smBGCs, *attP* site, and site-specific integrase can be inserted into the *attB* sites in the genome. While the CRISPR/Cas-based direct smBGC insertion method requires the preparation of editing templates for HDR, the *attB* insertion method is advantageous in that an engineered strain with multiple *attB* sites can be readily utilized without additional labor. However, the major drawback of an increase in the smBGC copy number is that silent smBGCs remain inactive regardless of the number of smBGCs in the genome; therefore, a readily applicable smBGC activation strategy is required. The CRISPRa strategy is an excellent option for *Streptomyces*, similar to the promoter knock-in strategy (Table 1). Although the expression of jadomycin B synthesis genes could be enhanced by exploiting CRISPRa in *S. venezuelae*, further optimization is required to effectively utilize CRISPRa in *Streptomyces*, as the efficiency of gene activation via CRISPRa in bacteria is heavily dependent on the targeting position and promoter type (Fontana et al., 2020; Ameruoso et al., 2022;). For this, dCas9 variants with expanded PAM sequences can be utilized (Fontana et al., 2020). To date, a versatile transcriptional activator, such as VP64 in the eukaryotic CRISPRa system, is absent in bacterial CRISPRa; thus, screening other transcriptional activators is required for the efficient application of CRISPRa in bacteria (Gilbert et al., 2014; Liu et al., 2019b).

**Fig. 3.**
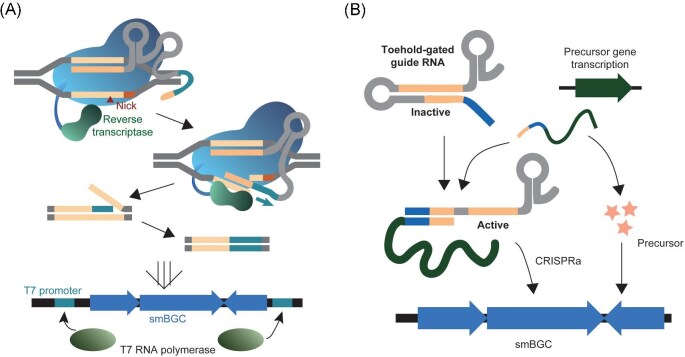
Potential application of CRISPR/Cas tools for secondary metabolite production. (A) Promoter replacement of secondary metabolite biosynthesis genes by exploiting prime editing. (B) Application of toehold-gated guide RNA for coordinating precursor supply and smBGC activation.

In addition to CRISPRa, other CRISPR/Cas-based genetic tools should be utilized to foster secondary metabolite research in *Streptomyces*. For instance, a new type of CRISPR/Cas-based genome editing tool called prime editing has been developed, which is advantageous for inserting short sequences (Anzalone et al., 2019). Prime editing consists of nCas9 fused with a reverse transcriptase and an extended guide RNA containing the primer-binding site and editing template for reverse transcription. Compared to CRISPR/Cas-based genome editing with the HDR pathway, prime editing is less toxic because a nick, rather than a DSB, is introduced in the genome. Additionally, constructing the plasmid is simplified as cloning of the donor template is not required. Given that T7 RNA polymerase can be utilized in *Streptomyces* for orthogonal expression, the insertion of a short T7 promoter sequence using a prime editing system would enable the expression of smBGCs, bypassing the host's transcriptional regulatory network (Fig. 3A) (Lussier et al., 2010; Wei et al., 2017).

For the timely control of gene expression, the CRISPR/Cas system regulated by inducible promoters could be effective for increasing secondary metabolite production. Recently, a cumate-inducible promoter was used to control the expression of both guide RNA and dCas9, and CRISPRi was found to be tightly regulated by the presence of cumates in various *Streptomyces* species (Bai & van Wezel, 2023). In addition, the activity of the CRISPR/Cas system can be autonomously regulated by utilizing toehold-gated guide RNA (Siu & Chen, 2019; Wang et al., 2023a), which activates the inactive modified guide RNA through toehold-mediated strand displacement by interacting with the host transcripts. The CRISPRi and CRISPRa systems can effectively coordinate the precursor supply with the onset of secondary metabolism by utilizing the transcripts of precursor supply genes as trigger strands (Fig. 3B). The integration of controllable CRISPRi and CRISPRa systems with precursor supply holds potential for significant advancements in secondary metabolite production.

### Exploiting CRISPR/Cas Systems for Heterologous Expression of Smbgcs

**Fig. 4.**
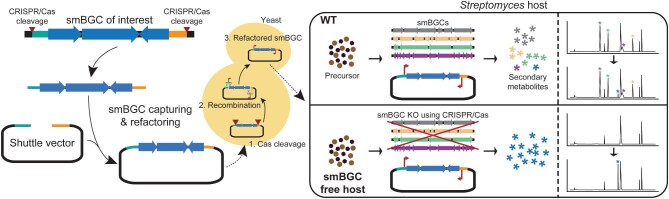
Heterologous expression of smBGCs harnessing the potential of the CRISPR/Cas system.

Although CRISPR/Cas systems have been successfully used in several *Streptomyces* species, the exploration of a large number of species is required for novel secondary metabolite discovery. Species-level heterogeneity often results in an inconsistent success rate or even the inability to achieve CRISPR/Cas-based genome editing for certain species (Cobb et al., 2015; Li et al., 2018; Yeo et al., 2019). Furthermore, some species may display slow growth rates in laboratory culture conditions or in the absence of proper genetic manipulation tools, further limiting *in vivo* CRISPR/Cas applications (Kieser et al., 2000). Additionally, many *Streptomyces* suffer from severe genetic instability, making it difficult to maintain engineered genotypes (Volff & Altenbuchner, 1998). Therefore, heterologous expression of smBGCs in well-established *Streptomyces* hosts is advantageous over the manipulation of each *Streptomyces* isolate. The CRISPR/Cas system can be utilized in diverse steps of smBGC heterologous expression, including the construction of a heterologous expression host, cloning of smBGC, and refactoring of smBGC with host-recognizable regulatory sequences (Fig. 4) (Kang et al., 2016; Fazal et al., 2020; Wu et al., 2020).

The construction of a host strain for the heterologous expression of smBGCs often involves the deletion of the host's smBGCs. The deletion of smBGCs proves beneficial for heterologous expression because of the increased cellular resources required for secondary metabolite synthesis (Fig. 4). In particular, PKSs commonly utilize malonyl- or methylmalonyl-CoA as a substrate, and the deletion of competing PKS-type BGCs can enhance the production of other polyketide-type secondary metabolites (Meng et al., 2016; Hwang et al., 2020). Furthermore, the smBGC-free host strain may provide a metabolically clean background for the detection of secondary metabolites synthesized from the introduced smBGC (Fig. 4) (Myronovskyi et al., 2018; Bu et al., 2019). Although only two *Streptomyces* strains, *S. albus* and *S. griseofuscus*, have been constructed using the CRISPR/Cas system, more smBGC-free heterologous expression host strains will be constructed in the near future (Fazal et al., 2020; Gren et al., 2021). In particular, large genomic deletions can induce severe defects in bacteria; thus, carefully supervised genome reduction with high-precision and time-saving genome editing by the CRISPR/Cas system is mandatory (Fig. 1A) (Dervyn et al., 2023). In addition, the utilization of CRISPR base editors to introduce premature stop codons to inactivate secondary metabolite synthesis genes enables extremely fast functional knockouts of smBGCs, harnessing the ease of multiplexity (Whitford et al., 2023b). However, further optimization is required to increase the efficiency of premature stop codon generation.

After the construction of a highly efficient host strain for heterologous expression, the smBGC of interest was cloned. However, most smBGCs of *Streptomyces* are quite large, often exceeding 100 kbp, and the cloning of large-sized DNA is the major bottleneck (Miao et al., 2005). In addition, the high GC content of the *Streptomyces* genome hampers the acquisition of whole smBGCs by PCR (McDowell et al., 1998). Although direct pathway cloning, comprising PCR amplification of partial smBGCs and assembly of PCR products, has been exploited to clone smBGCs, its application has been limited to relatively short DNA fragments (∼25 kbp) (Greunke et al., 2018). Cloning of longer DNA requires screening target smBGCs from cosmid, fosmid, or BAC libraries bearing random genomic DNA fragments, which requires considerable labor and time (Miao et al., 2005). To increase the selectivity of the target genomic fragment, homology-dependent cloning methods, particularly transformation-associated recombination (TAR) cloning, have been utilized (Zhang et al., 2019). However, obtaining the precise smBGC region is a bottleneck. To overcome these limitations, the CRISPR/Cas system can be used *in vitro* to obtain smBGCs directly from the genome of *Streptomyces* (Fig. 4) (Jiang et al., 2015). The major obstacle to capturing large DNA fragments *in vitro* is that genomic DNA is prone to shearing (Moore et al., 1999). To minimize the shearing stress of genomic DNA during extraction and cleavage by the CRISPR/Cas system, the cells are immobilized in agarose plugs, and the lysis of the cells and cleavage of the genomic DNA by CRISPR/Cas9 is processed in a gel (Jiang et al., 2015). Cas9 cleavage generates a blunt-end product; thus, Gibson assembly was used to ligate the DNA fragment into the cloning vector. To increase cloning efficiency, Cas12a, which generates sticky ends, can be used as an advanced alternative to Cas9 (Liang et al., 2022). However, *in vitro* assembly of large DNA fragments may generate undesired by-products, such as DNA concatemers (Dugaiczyk et al., 1975). To further elevate the efficiency of smBGC cloning, the smBGC fragment from Cas12a cleavage can be circularized via *in vivo* Cre-*lox* recombination (Enghiad et al., 2021). First, two *lox*P sites are introduced at the both ends of the target smBGC fragment, along with either origin or selection marker required for maintaining the plasmid. The resulting linear DNA is then transformed into *Escherichia coli* harboring Cre-*lox* recombination system for *in vivo* circularization of the smBGC containing plasmid. This approach facilitated cloning of 47 different smBGCs ranging from 10 to 113 kbp.

Heterologous expression of smBGCs can be advantageous compared to engineering native-producing organisms, in that the complex transcriptional regulation of natural producers can be bypassed (Xu & Wright, 2019). However, the genetic background of the expression host may not be suitable for the transcription of foreign smBGC; thus, appropriate refactoring of the promoters for the biosynthesis of genes may be required. Scarless and multiplexed DNA editing of nearly unrestricted target positions using the CRISPR/Cas system has enormous utility for refactoring smBGCs. In addition, the CRISPR/Cas system can be directly utilized to engineer smBGCs, especially by refactoring promoters in a heterologous expression host, which is well suited for CRISPR/Cas-mediated genetic engineering.

## Concluding Remarks and Future Perspectives

This review briefly summarizes the broad range of CRISPR/Cas applications in *Streptomyces* for natural product discovery and production. The genetic outcome of CRISPR/Cas-based engineering is the same as that of conventional double-crossover mediated genome editing. However, the dramatically reduced time and labor required for genetic manipulation opens the possibility of investigating an enormous amount of uncharacterized smBGCs and relieving the imbalance between smBGCs and secondary metabolite discovery (Kieser et al., 2000; Lee et al., 2020a). However, there remain obstacles and improvements for CRISPR/Cas applications in *Streptomyces*. As *Streptomyces* represents one of the largest genera of bacteria, it is difficult to establish common genetic engineering tools for all species, and every step in genetic manipulation may require optimization for each species (Kieser et al., 2000). At present, only three types of CRISPR/Cas systems have been demonstrated for the genetic engineering of *Streptomyces*, and other types of CRISPR/Cas systems could be exploited to overcome the species-level heterogeneity of genetic engineering (Koonin et al., 2017). In addition to expanding the available CRISPR/Cas systems, heterologous expression in genetically tractable and metabolically clean *Streptomyces* hosts can also be exploited (Fazal et al., 2020). In particular, the use of a chassis strain and a uniform genetic manipulation protocol for heterologous expression enables the automation of experimental procedures (Yuan et al., 2022). Although the CRISPR/Cas system dramatically reduced the time required for *Streptomyces* engineering, engineering smBGCs remains a bottleneck for secondary metabolite discovery compared to *Streptomyces* isolation and smBGC prediction (Lee et al., 2020a). In this regard, the automation of experimental procedures, including cloning and refactoring of smBGCs, production in heterologous expression hosts, and identification of the synthesized molecule, is urgently needed to enable extensive investigation of uncharacterized smBGCs (Yuan et al., 2022, 2023). Recently, new genetic engineering tools that harness the precise DNA-targeting ability of the CRISPR/Cas system have been developed and applied in *Streptomyces*. The CRISPR/Cas system fosters natural product research and is poised to become the basis for the a potential second ‘golden era’ in antibiotic discovery.
